# Predicting copper leaching from slag: an interpretable machine learning approach under oxidative sulfuric acid conditions

**DOI:** 10.1039/d6ra01571a

**Published:** 2026-04-13

**Authors:** Sung-Jin Kim, Song-Sae Kang, Kyong-Nam Pae, Song-Il Pak, Hyon-Il Jo, Ryong-Jin Kim

**Affiliations:** a Faculty of Materials Science, Kim Il Sung University Pyongyang 497335 Democratic People's Republic of Korea ksj1223@163.com; b Faculty of Information Engineering, Pyongyang HanTokSu University of Light Industry Pyongyang 999093 Democratic People's Republic of Korea; c Faculty of Physical Engineering, Kim Chaek University of Technology Pyongyang 950003 Democratic People's Republic of Korea

## Abstract

Efficient recovery of copper from metallurgical waste is essential for sustainable resource utilization. This study develops an interpretable machine learning framework to predict copper leaching efficiency from copper slag under oxidative sulfuric acid conditions. A comprehensive multi-source dataset comprising 465 experimentally reported data points collected from multiple peer-reviewed studies was compiled from peer-reviewed literature. Four algorithms, Random Forest, Support Vector Regression, XGBoost, and LightGBM, were systematically optimized using 10-fold cross-validation. XGBoost demonstrated superior predictive performance with *R*^2^ = 0.9794, RMSE = 3.4757, and MAE = 2.3442 on the test set. SHAP-based interpretability analysis revealed that operational parameters, particularly leaching time, acid concentration, and temperature, exert dominant influence over copper extraction, while compositional variables such as Si, S, and Al show limited direct contribution within the investigated dataset range. The nonlinear trends identified are consistent with shrinking-core kinetics and diffusion-controlled mechanisms. External validation using independent literature datasets confirmed robust generalization capability. The proposed framework provides quantitative guidance for process optimization and offers a practical tool for enhancing sustainable metal recovery from metallurgical waste.

## Abstract 2

An interpretable XGBoost model predicts copper leaching efficiency from slag with *R*^2^ = 0.9794. SHAP analysis shows time, acid, and temperature dominate, enabling quantitative process optimization for sustainable recovery.

## Graphical abstract

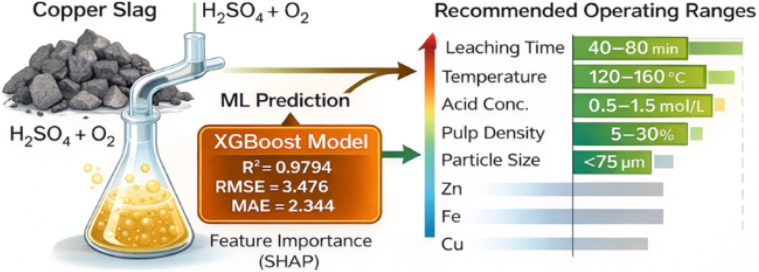

## 1 Introduction

Copper slag is a major by-product generated during pyrometallurgical copper production and contains residual valuable metals, particularly copper, along with iron-rich silicate phases.^1–3^ With the increasing emphasis on sustainable resource utilization and waste valorization, the recovery of copper from slag has attracted considerable attention.^4^ Hydrometallurgical treatment using sulfuric acid under oxidative conditions is widely regarded as a promising route due to its operational flexibility and relatively mild processing requirements.^5^ However, copper leaching from slag is governed by complex interactions among slag composition, reaction temperature, acid concentration, oxygen pressure, and particle size, making process optimization challenging.^6^

Traditional investigations of copper slag leaching primarily rely on controlled laboratory experiments to evaluate the influence of individual process parameters.^7–9^ While these studies provide valuable mechanistic insights, they often focus on limited experimental ranges and isolated variable effects.^10^ The inherent multivariable coupling in leaching systems, including reaction–diffusion interactions and compositional heterogeneity, is difficult to fully capture using conventional regression or empirical modeling approaches.^11,12^ Moreover, inconsistencies in experimental conditions across published studies hinder the development of generalized predictive relationships.

Machine learning (ML) techniques have recently emerged as powerful tools for modeling complex nonlinear systems in metallurgical and hydrometallurgical processes.^13–17^ Algorithms such as Random Forest, gradient boosting methods, and support vector regression have demonstrated strong predictive capability in various materials-processing applications.^11,18,19^

Recently, Weng *et al.* (2025) reported a machine-learning-assisted framework for copper slag acid leaching, identifying Random Forest as the optimal predictive model and demonstrating the feasibility of literature-derived data integration for process optimization.^20^ Their work represents an important step toward data-driven hydrometallurgy. Building on this foundation, the present study extends previous machine learning applications by employing a larger dataset and an optimized predictive model. A total of 465 data points were compiled from multiple literature sources, substantially larger than the 265 data points used in ref. 20. Based on this dataset, the optimized XGBoost model achieved *R*^2^ = 0.9794, RMSE = 3.4757, and MAE = 2.3442 on the test set, compared to *R*^2^ = 0.91, RMSE = 7.492, and MAE = 5.681 reported in the same study.

Nevertheless, several aspects remain to be further explored. The dataset size in previous studies remains relatively moderate, and interpretability analysis has largely relied on conventional feature-importance metrics and partial dependence plots.^21^ More importantly, systematic validation using independent literature sources not involved in model training has not been rigorously implemented. These factors may limit model generalization capability and reduce mechanistic transparency in practical applications.

To address these challenges, the present study develops an expanded and interpretable machine-learning framework for predicting copper leaching efficiency from copper slag under sulfuric acid oxidative conditions. A comprehensive multi-source dataset comprising 465 experimental data points was compiled from peer-reviewed literature, covering broad compositional and operational ranges. Four supervised learning algorithms, including Random Forest, Support Vector Regression, XGBoost, and LightGBM, were systematically optimized using 10-fold cross-validation and comparatively evaluated.

To enhance mechanistic interpretability, SHapley Additive exPlanations (SHAP) were employed to quantify both global and local feature contributions and to elucidate nonlinear interactions among variables. Furthermore, independent datasets obtained from separate literature sources not included in model training were utilized to rigorously assess model robustness and generalization capability.

By integrating large-scale literature data harmonization, advanced ensemble learning, SHAP-based interpretation, and external validation, this work aims to establish a predictive and mechanistically meaningful framework for copper slag hydrometallurgy. The proposed approach not only improves predictive accuracy but also provides actionable insights for process optimization and preliminary industrial design.

## 2 Methods

### 2.1 Dataset construction and preprocessing

Experimental data for copper leaching from copper slag were collected from peer-reviewed journal articles focusing on sulfuric acid oxidative leaching systems.^7,20,22–27^ A comprehensive literature survey was conducted to identify studies reporting quantitative information on slag chemical composition, operating conditions, and copper leaching efficiency. Only studies providing complete datasets including both input variables and the corresponding leaching efficiency were retained.

To ensure mechanistic consistency, studies involving chloride systems, bioleaching processes, reductive environments, or multi-acid mixed systems were excluded. Only sulfuric acid leaching under oxidative conditions was considered, thereby minimizing variability in reaction pathways and maintaining physicochemical comparability across the compiled data.

After systematic screening and filtering, a total of 465 valid experimental data points were obtained. The final dataset comprises six compositional variables of copper slag (Cu, Fe, Si, S, Zn, and Al contents, wt%) and six operational parameters: particle size (PS, µm), leaching time (LT, min), oxygen pressure (OP, kPa), pulp density (PD, g L^−1^), temperature (*T*, °C), and sulfuric acid concentration (AC, mol L^−1^). The target variable is copper leaching efficiency (LE, %).

Although stirring speed is recognized as an important hydrodynamic parameter affecting external mass transfer and diffusion behavior during hydrometallurgical leaching processes, it was not included as an input variable in the present modeling framework. In heterogeneous leaching systems, agitation influences the thickness of the liquid boundary layer surrounding solid particles and therefore may affect the external mass transfer coefficient. However, in the literature sources used to construct the present dataset, stirring speed was not consistently reported or standardized across studies. Incorporating this variable would therefore have significantly reduced the number of usable data points and weakened the statistical representativeness of the compiled dataset.

Furthermore, most of the experimental studies included in this dataset were conducted under sufficiently vigorous agitation conditions intended to minimize external diffusion limitations and ensure homogeneous suspension of slag particles. Under such conditions, leaching kinetics are generally dominated by intrinsic reaction processes or internal diffusion within product layers rather than by external film diffusion. Consequently, the omission of stirring speed is not expected to fundamentally alter the predictive relationships captured by the machine learning models within the investigated parameter space. Nevertheless, the potential influence of hydrodynamic conditions should be considered when extrapolating the model predictions to industrial-scale reactors or systems operating under different agitation regimes.

In most of the collected studies, particle size was reported as a size interval (*e.g.*, 38–75 µm) rather than a single representative value. To ensure numerical consistency and enable quantitative modeling, the arithmetic mean of each reported size range was adopted as the representative particle size. This treatment assumes an approximately uniform particle size distribution within the specified interval, which is a common approximation in leaching studies. Although this simplification may introduce minor uncertainty associated with intra-range distribution effects, it allows preservation of a large and diverse dataset while maintaining modeling feasibility.

All variables were harmonized to ensure unit consistency. Acid concentrations originally reported in g L^−1^ were converted into mol L^−1^ based on molar mass; oxygen pressure values were standardized to kPa; and temperature values were unified in °C. Where necessary, weight percentages were converted into consistent wt% representation. This harmonization procedure minimizes systematic bias arising from inconsistent reporting formats across different literature sources.

Entries with incomplete information for any of the selected variables were excluded to avoid uncertainty introduced by data imputation. Outliers were evaluated using the interquartile range (IQR) method. However, extreme values were retained if they corresponded to experimentally valid conditions reported in the original studies, ensuring that physically meaningful high-temperature or high-pressure conditions were not artificially removed.

**Fig. 1:**
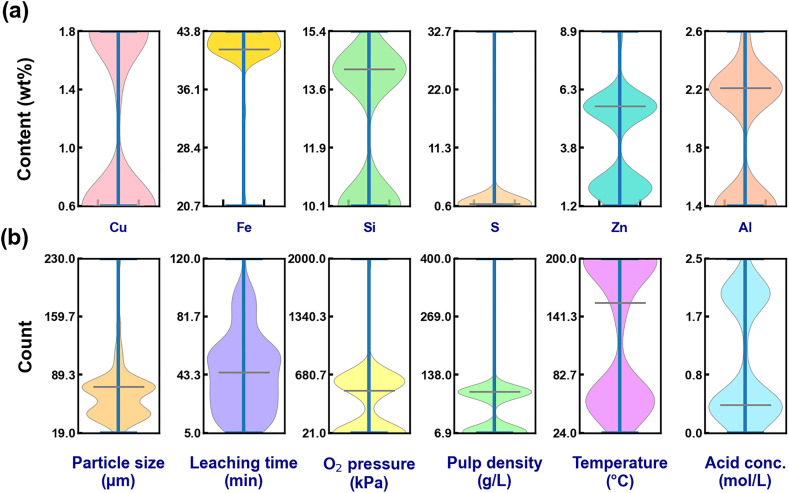
Violin boxplots showing (a) distribution of elements (Cu, Fe, Si, S, Zn, Al) in used copper slags, and (b) operational parameters (particle size, leaching time, oxygen pressure, pulp density, temperature, H_2_SO_4_ concentration) during copper slag leaching.

Descriptive statistics of the processed dataset are summarized in Table 1, and variable distributions are illustrated in Fig. 1. The dataset exhibits broad distributions in oxygen pressure (21–2000 kPa), temperature (24–200 °C), pulp density (6.93–400 g L^−1^), and leaching time (5–120 min), indicating substantial variability in experimental conditions. Such diversity enhances the robustness and generalization capability of the developed machine learning models.

**Table 1:** The data details used for machine leaching

	Cu (%)	Fe (%)	Si (%)	S (%)	Zn (%)	Al (%)	PS (µm)	LT (min)	OP (kPa)	PD (g L^−1^)	T (°C)	AC (mol L^−1^)	LE (%)
Count	465	465	465	465	465	465	465	465	465	465	465	465	465
Mean	1.18	41.47	12.42	1.88	4.06	1.91	69.01	45.41	364.81	59.63	127.93	0.99	64.40
Std	0.59	4.35	2.08	5.02	2.01	0.43	35.75	26.19	383.50	49.14	69.31	0.82	23.51
Min	0.64	20.70	10.12	0.59	1.20	1.43	19.00	5.00	21.00	6.93	24.00	0.00	8.00
25%	0.64	41.36	10.12	0.98	2.02	1.43	41.50	25.00	21.00	10.04	60.00	0.40	48.51
50%	0.64	41.36	14.23	0.98	5.60	2.24	75.00	45.00	500.00	100.00	155.00	0.40	68.35
75%	1.84	43.76	14.23	1.21	5.60	2.24	75.00	60.00	600.00	100.00	200.00	2.00	84.03
Max	1.84	43.76	15.37	32.7	8.9	2.63	230.00	120.00	2000.00	400.00	200.00	2.50	99.00

For model development, the dataset was randomly divided into training (80%) and testing (20%) subsets. Hyperparameter optimization was conducted using 10-fold cross-validation to improve statistical reliability and mitigate overfitting. A fixed random seed was applied to ensure reproducibility of all modeling results. For tree-based models (RF, XGBoost, and LightGBM), raw feature values were directly used without scaling. For the SVR model, input features were standardized using Z-score normalization to enhance numerical stability and convergence performance.

Although the dataset size is moderate compared to large industrial datasets, it represents one of the most comprehensive harmonized collections of copper slag leaching data currently available in open literature.

### 2.2 Input and output variables

The selection of input variables was guided by physicochemical relevance to copper leaching behavior and data availability across the compiled literature sources. The input features were categorized into two groups: (i) intrinsic compositional variables of the copper slag and (ii) operational parameters governing leaching kinetics.

The compositional variables include Cu, Fe, Si, S, Zn, and Al contents (wt%). These variables reflect the mineralogical and chemical characteristics of the slag matrix. The Cu content represents the primary recoverable metal phase and directly affects the theoretical leaching capacity. Fe content is associated with fayalite and magnetite phases, which influence slag structure and acid consumption. Si content is related to silicate network stability and may affect slag dissolution resistance. Sulfur content can indicate the presence of sulfide phases, potentially altering oxidative leaching behavior. Zn and Al are considered secondary metallic components that may influence solution chemistry and acid consumption during leaching.

The operational parameters include particle size, leaching time, oxygen pressure, pulp density, temperature, and sulfuric acid concentration. Particle size affects the available reactive surface area and internal diffusion distance. Leaching time reflects the progression of reaction kinetics. Oxygen pressure governs oxidative conditions and influences redox reactions, particularly for sulfide-containing phases. Pulp density controls solid–liquid ratio and mass transfer behavior. Temperature affects both reaction rate and diffusion coefficients. Acid concentration determines proton availability and dissolution driving force.

The target variable is copper leaching efficiency (%), defined as the percentage of copper dissolved relative to its initial content in the slag. This metric directly reflects process performance and recovery efficiency.

**Fig. 2:**
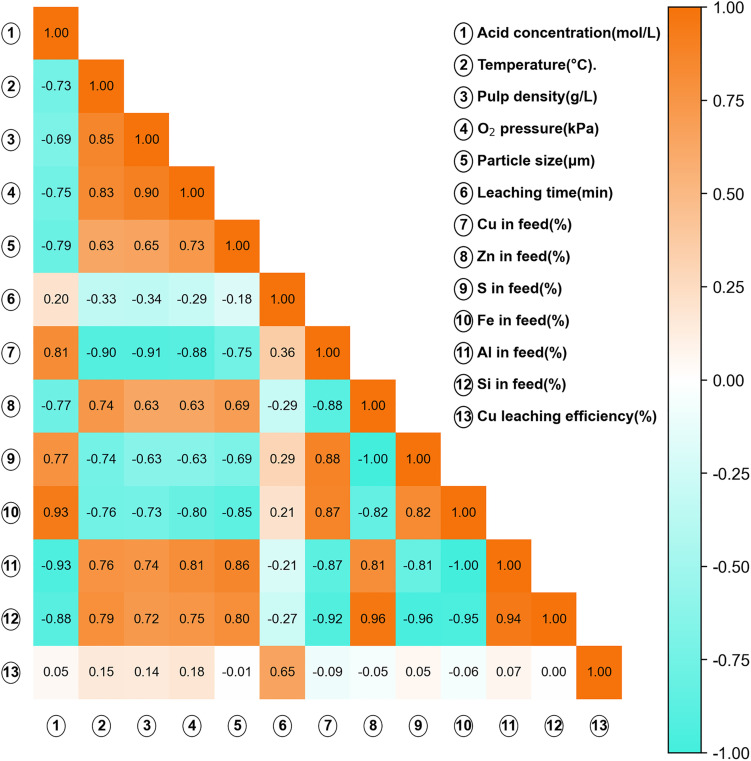
Spearman correlation coefficient between all input and output variables.

Although certain variables may exhibit statistical correlations (as illustrated in Fig. 2), no feature elimination was performed prior to model development. Tree-based models inherently handle multicollinearity, and retaining all physically meaningful variables allows preservation of mechanistic interpretability. The feature importance and interaction effects were subsequently analyzed using SHAP to further elucidate variable contributions.

The chosen input–output framework thus integrates intrinsic material characteristics and key thermodynamic-kinetic operating parameters, enabling development of predictive yet physically interpretable machine learning models for copper slag leaching.

### 2.3 Machine learning algorithms

To develop a robust and interpretable predictive framework for copper leaching efficiency, four supervised machine learning algorithms were employed: Random Forest (RF),^28^ Support Vector Regression (SVR),^29^ Extreme Gradient Boosting (XGBoost),^30^ and Light Gradient Boosting Machine (LightGBM).^31^ These models were selected to represent distinct learning paradigms, including bagging-based ensemble learning, kernel-based regression, and gradient boosting frameworks.

#### 2.3.1 Random Forest (RF)

RF is an ensemble learning method based on bootstrap aggregation (bagging) of multiple decision trees. Each tree is trained using a random subset of samples and features, and the final prediction is obtained by averaging individual tree outputs. This structure enhances model stability and reduces variance compared to a single decision tree.

In previous studies addressing copper slag leaching and related hydrometallurgical processes, RF has been reported as one of the most suitable models for capturing nonlinear relationships between compositional factors and process variables.^20^ Its robustness to multicollinearity and noise makes it particularly appropriate for literature-compiled datasets with heterogeneous experimental conditions. In this study, key hyperparameters including the number of trees (n_estimators) and maximum tree depth (max_depth) were optimized.

#### 2.3.2 Support Vector Regression (SVR)

SVR is a kernel-based regression method derived from statistical learning theory. It determines an optimal regression hyperplane by minimizing structural risk within an *ε*-insensitive loss framework. The radial basis function (RBF) kernel was adopted to capture nonlinear relationships between variables. Hyperparameters including the regularization parameter (*C*), kernel width (*γ*), and epsilon (*ε*) were tuned using cross-validation. Although SVR is more sensitive to feature scaling and dataset size, it provides a theoretically rigorous nonlinear regression baseline for comparison.

#### 2.3.3 Extreme Gradient Boosting (XGBoost)

XGBoost is an advanced gradient boosting algorithm that sequentially constructs decision trees by minimizing a regularized objective function. It incorporates second-order gradient optimization, shrinkage, and regularization mechanisms to prevent overfitting and enhance generalization performance.

In recent years, XGBoost has been increasingly adopted in hydrometallurgical and leaching-related studies due to its strong nonlinear learning capability and superior predictive accuracy compared to traditional ensemble models.^32–34^ Its ability to capture complex interactions among compositional and operational variables makes it particularly suitable for multi-factor copper slag leaching systems. In this work, hyperparameters such as maximum tree depth (max_depth), learning rate (*η*), and number of estimators (n_estimators) were optimized using 10-fold cross-validation.

#### 2.3.4 Light Gradient Boosting Machine (LightGBM)

LightGBM is a gradient boosting framework characterized by leaf-wise tree growth and high computational efficiency. Compared to traditional level-wise boosting algorithms, LightGBM can reduce training loss more efficiently while maintaining scalability. Key hyperparameters including number of leaves (num_leaves), maximum depth (max_depth), and number of estimators (n_estimators) were tuned to achieve optimal performance.

All models were implemented using Python-based machine learning libraries. Hyperparameter optimization was performed *via* grid search combined with 10-fold cross-validation on the training dataset. The final predictive performance was evaluated using the independent testing subset.

By incorporating bagging-based, kernel-based, and boosting-based algorithms, the modeling framework enables systematic performance comparison and ensures robustness of the predictive conclusions.

### 2.4 Model evaluation and interpretation methods

To comprehensively evaluate the predictive performance of the developed machine learning models, three widely adopted regression metrics were employed: coefficient of determination (*R*^2^), root mean square error (RMSE), and mean absolute error (MAE).^35^

The coefficient of determination (*R*^2^) measures the proportion of variance in the observed data explained by the model and is defined as:1
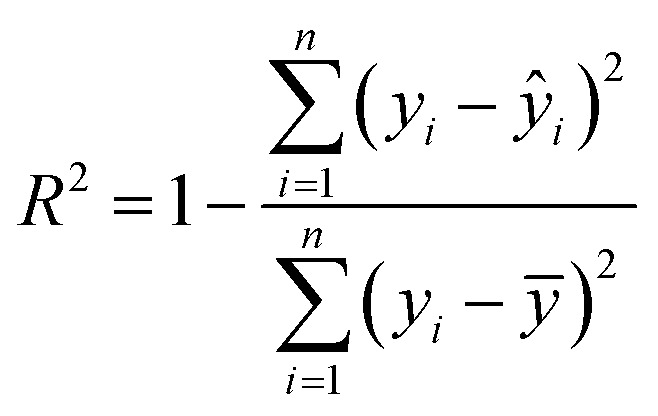
where *y*_*i*_ is the experimental value, *ŷ*_*i*_ is the predicted value, and *ȳ* is the mean of experimental values.

RMSE quantifies the standard deviation of prediction errors and emphasizes large deviations:2
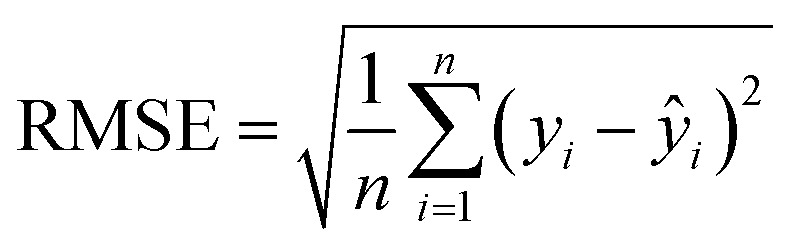


MAE represents the average absolute deviation between predicted and observed values:3
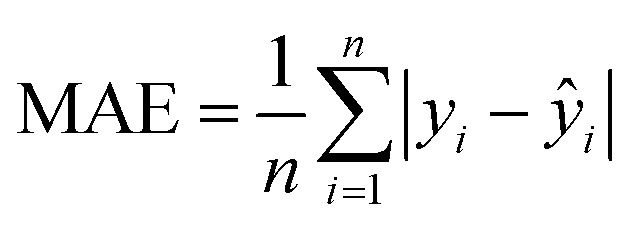


While *R*^2^ evaluates overall goodness-of-fit, RMSE penalizes large errors more heavily, and MAE provides a scale-consistent measure of average prediction deviation. The combined use of these metrics ensures balanced performance assessment.

To mitigate overfitting, hyperparameter optimization was conducted using 10-fold cross-validation on the training dataset. Model performance was subsequently evaluated using an independent testing subset not involved in model training or tuning. This separation ensures an unbiased estimation of predictive capability.

Beyond predictive accuracy, model interpretability was investigated using SHapley Additive exPlanations (SHAP).^36^ SHAP values are derived from cooperative game theory and quantify the marginal contribution of each feature to the model prediction. Global feature importance was evaluated through mean absolute SHAP values, allowing ranking of influential variables. Additionally, SHAP dependence plots were employed to explore nonlinear relationships and interaction effects between input variables and copper leaching efficiency.

Partial dependence behavior was further analyzed to examine how variations in individual features influence predicted leaching efficiency while averaging out other variables. This approach enables identification of threshold effects, nonlinear transitions, and potential synergistic interactions among operational parameters.

To assess model generalization, additional validation analyses were performed under distinct operating condition subsets. This case-based evaluation examines the robustness of the selected optimal model when applied to different compositional or process regimes, thereby strengthening confidence in its practical applicability.

Through the integration of quantitative performance metrics and interpretable machine learning techniques, the evaluation framework not only identifies the most accurate predictive model but also provides mechanistic insights into the governing factors of copper slag leaching.

## 3 Results and discussion

### 3.1 Statistical analysis of pre-processed dataset

Descriptive statistical characteristics of the compiled dataset are summarized in Table 1, and the distribution profiles of input and output variables are illustrated in Fig. 1. The dataset consists of 465 data points, covering a broad range of compositional and operational conditions, thereby providing a statistically meaningful basis for machine learning modeling.

The copper content in the slag varies from 0.64% to 1.84%, with a mean value of 1.18% and a relatively moderate standard deviation (0.59%). This indicates that the dataset encompasses both low-grade and relatively enriched copper slags. Iron content exhibits a narrower distribution (20.70–43.76%, mean 41.47%), reflecting the dominance of iron-bearing phases such as fayalite in copper slag matrices. Silicon content ranges from 10.12% to 15.37%, suggesting variability in silicate network structure that may influence dissolution resistance.

Sulfur content presents a comparatively wider spread (0.59–32.7%), although the interquartile range indicates that most samples contain relatively low sulfur levels. This variability may reflect differences in slag production routes and residual sulfide phases. Zinc (1.20–8.9%) and aluminum (1.43–2.63%) contents exhibit moderate dispersion, potentially influencing acid consumption and secondary dissolution reactions.

The violin plots in Fig. 1 reveal that several compositional variables exhibit skewed distributions rather than symmetric normal distributions. Such non-normality supports the use of nonlinear machine learning algorithms capable of handling complex feature-response relationships.

Operational parameters exhibit substantial variability across studies. Particle size ranges from 19 to 230 µm (mean 69.01 µm), reflecting differences in grinding conditions and liberation levels. Leaching time varies from 5 to 120 min (mean 45.41 min), covering both early-stage and near-equilibrium dissolution regimes.

Oxygen pressure displays a particularly broad distribution (21–2000 kPa), with a high standard deviation (383.50 kPa), indicating that both atmospheric and high-pressure oxidative leaching conditions are represented. Temperature ranges from 24 to 200 °C (mean 127.93 °C), spanning mild to hydrothermal regimes. Pulp density varies significantly (6.93–400 g L^−1^), suggesting diverse solid–liquid interaction intensities across the dataset. Acid concentration covers 0–2.50 mol L^−1^ (mean 0.99 mol L^−1^), indicating both dilute and moderately concentrated leaching environments.

The wide distribution ranges of these operational parameters, as visualized in Fig. 1, demonstrate substantial experimental diversity. Such variability enhances the robustness of the predictive modeling framework by exposing algorithms to a wide range of reaction regimes.

Copper leaching efficiency (LE) ranges from 8% to 99%, with a mean value of 64.40% and a standard deviation of 23.51%. The broad distribution of LE indicates that the dataset includes both low-efficiency and near-complete extraction scenarios. This diversity is advantageous for regression modeling, as it prevents bias toward a narrow operational window and improves the ability to learn nonlinear trends.

Spearman correlation coefficients between input variables and copper leaching efficiency are presented in Fig. 2. The nonparametric Spearman method was adopted due to the non-normal distribution characteristics observed in several variables.

Operational parameters such as temperature, acid concentration, and leaching time generally exhibit positive correlations with leaching efficiency, consistent with reaction kinetics and thermodynamic expectations. Oxygen pressure also shows a positive association, reflecting the role of oxidative conditions in promoting copper dissolution.

Conversely, particle size tends to exhibit a negative correlation with leaching efficiency, which is consistent with surface-area-controlled and diffusion-limited dissolution mechanisms. Among compositional variables, copper content shows a positive correlation with LE, whereas high silica content may display weaker or slightly negative correlations due to increased structural stability of silicate matrices.

It is noteworthy that certain input variables exhibit intercorrelations, particularly among operational parameters. However, no extreme multicollinearity was observed that would necessitate feature elimination. Moreover, tree-based models employed in this study are inherently robust to moderate multicollinearity, and retaining all physically meaningful variables preserves interpretability for subsequent SHAP-based analysis.

Overall, the statistical analysis indicates that the compiled dataset covers wide compositional and operational regimes, with substantial variability in both input and output variables. The presence of nonlinear distributions and moderate inter-feature correlations further justifies the application of nonlinear ensemble learning algorithms.

The diversity and scale of the dataset provide a solid statistical foundation for developing generalizable and interpretable predictive models for copper slag leaching efficiency.

### 3.2 Model optimization and performance comparison

**Fig. 3:**
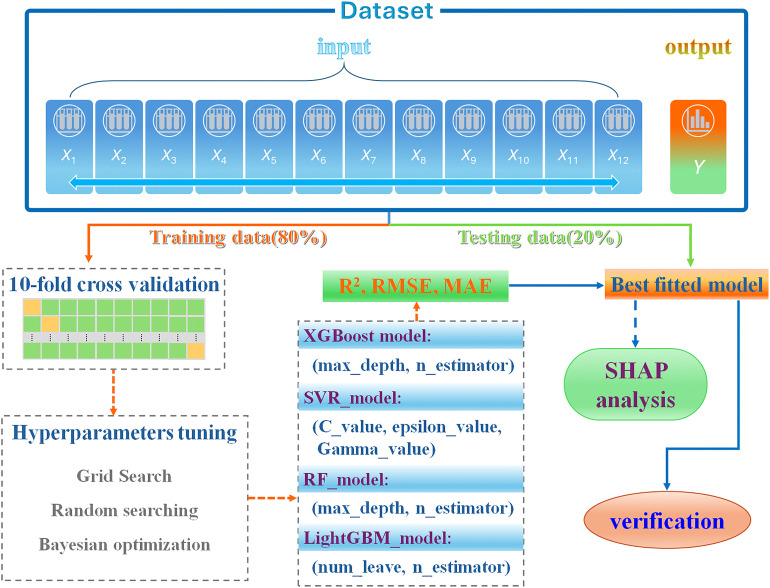
Flowchart for model training. Testing, and evaluation. *R*^2^-coefficient of determination; RMSE-root mean square error; MAE-mean absolute error.

The overall modeling workflow is illustrated in Fig. 3. The dataset was divided into training and independent test subsets, and hyperparameter optimization was performed within the training set using 10-fold cross-validation. The 10-fold cross-validation results showed low standard deviation across folds, indicating stable learning behavior and limited variance. Model performance was evaluated on the test set using *R*^2^, RMSE, and MAE metrics. For each algorithm, key hyperparameters were systematically tuned within predefined ranges to achieve optimal predictive performance.

**Fig. 4:**
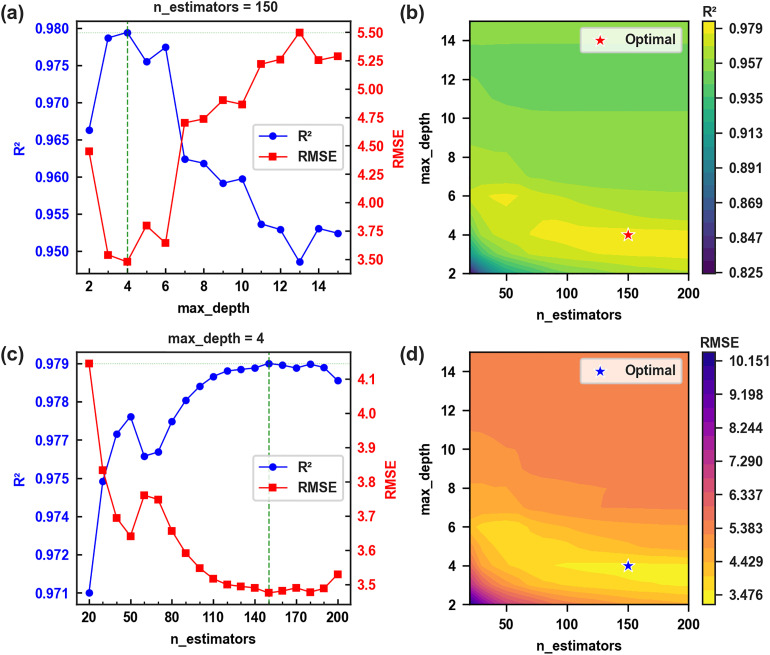
(a) Optimization for the parameter of max_depth of the XGBoost model; (b) optimization for the parameter of n_estimator of the XGBoost model; (c) dependence of *R*^2^ with the max_depth and n_estimators; (d) dependence of RMSE with the max_depth and n_estimators.

Among the four evaluated models, XGBoost exhibited the most robust and stable optimization behavior (Fig. 4). The response surface analysis revealed a broad performance plateau across varying combinations of max_depth and n_estimators, indicating limited sensitivity to moderate hyperparameter deviations. This stability reflects the effectiveness of the regularized objective function and second-order gradient optimization in controlling model complexity while preserving nonlinear learning capacity. The relatively smooth performance gradients further suggest enhanced robustness under practical tuning constraints.

In contrast, Random Forest showed diminishing performance gains beyond a threshold number of estimators, consistent with the variance-reduction mechanism inherent to bagging ensembles (Fig. S1). Although competitive in accuracy, its ability to capture complex nonlinear feature interactions remained inferior to boosting-based approaches.

SVR demonstrated pronounced sensitivity to hyperparameter selection (Fig. S2). Large values of *C* increased variance and overfitting risk, whereas small *γ* values limited nonlinear representation capability. Despite careful grid optimization, the narrow optimal region reduced robustness across parameter configurations.

LightGBM (Fig. S3) achieved rapid performance improvement with increasing tree complexity due to its leaf-wise growth strategy. However, excessive depth led to diminishing returns and elevated overfitting risk, particularly under limited data conditions, resulting in higher variance compared to XGBoost.

The hyperparameter search ranges and corresponding optimal values obtained through grid-search optimization are summarized in Table S1.

The training times of the four machine learning models under identical hardware and software conditions are summarized in Table S2, demonstrating the superior computational efficiency of LightGBM relative to XGBoost, Random Forest, and SVR.

**Fig. 5:**
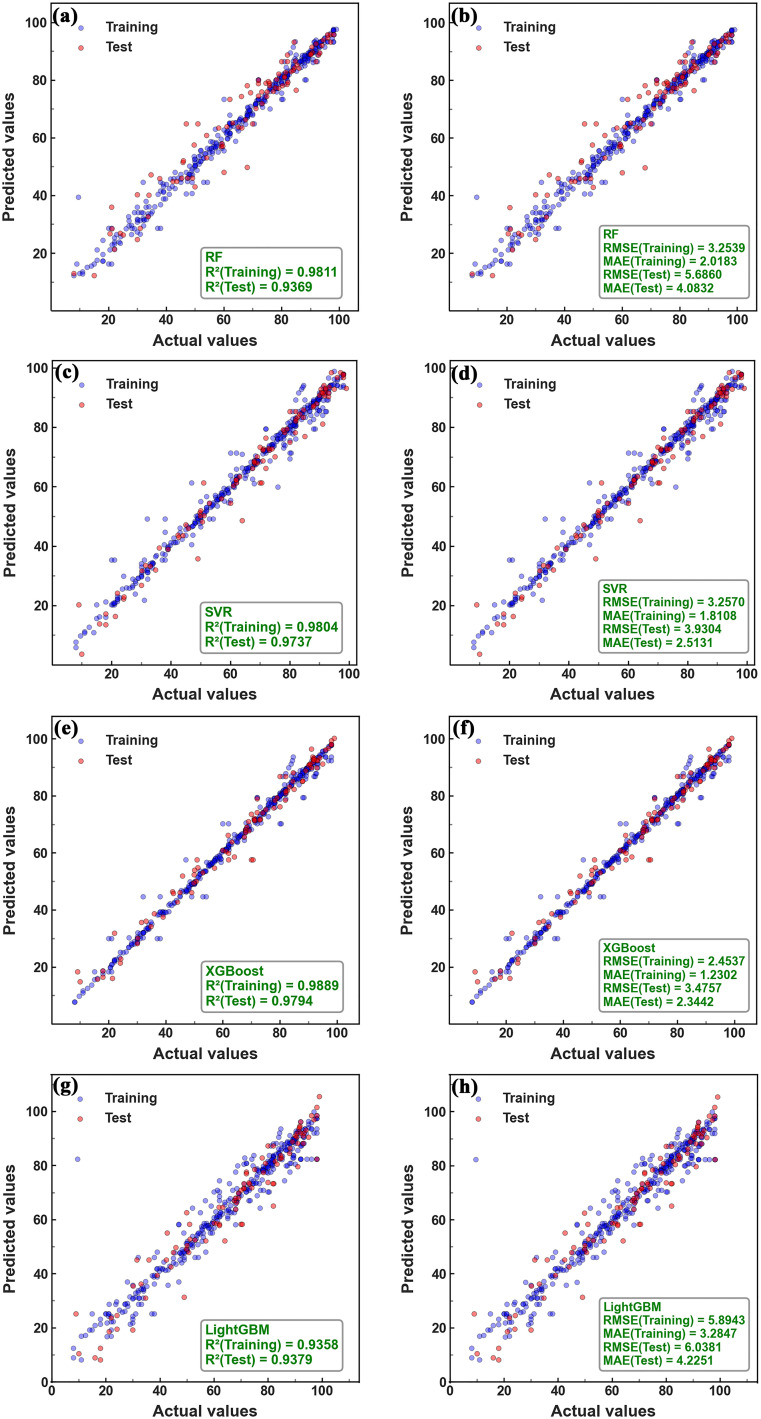
Copper leaching efficiency predicted by various models: (a) *R*^2^ of RF, (b) RMSE and MAE of RF, (c) *R*^2^ of SVR, (d) RMSE and MAE of SVR, (e) *R*^2^ of XGBoost, (f) RMSE and MAE of XGBoost, (g) *R*^2^ of LightGBM, (h) RMSE and MAE of LightGBM.

Performance comparison across optimized models (Fig. 5) confirmed that XGBoost achieved the highest *R*^2^ and lowest RMSE and MAE on the test dataset, demonstrating superior predictive accuracy and generalization capability. Considering its consistent performance across evaluation metrics and stable convergence characteristics during optimization, XGBoost was selected as the baseline model for subsequent SHAP-based interpretability analysis. Its structural capacity to model high-order feature interactions further supports this selection for complex multi-factor leaching systems.

The predictive performance of the four optimized models is summarized in Fig. 5. For clarity, Fig. 5a–h compares *R*^2^, RMSE, and MAE values across RF, SVR, XGBoost, and LightGBM models.

Among the evaluated algorithms, XGBoost achieved the highest *R*^2^ and the lowest RMSE and MAE on the testing dataset, demonstrating superior predictive accuracy and generalization capability. SVR exhibited competitive accuracy but yielded slightly lower *R*^2^ and somewhat higher RMSE and MAE values compared to XGBoost. Random Forest, which has been reported in previous studies as a relatively high-performance model for copper slag leaching modeling, showed lower predictive performance than both XGBoost and SVR in this study. LightGBM exhibited comparatively lower predictive performance under the present dataset conditions.

These performance discrepancies can be attributed to the structural characteristics of each algorithm. XGBoost effectively captures complex nonlinear relationships within the data while rigorously controlling overfitting through its regularized objective function and second-order gradient optimization. The balanced bias-variance trade-off achieved by XGBoost contributes to its superior generalization performance. SVR is capable of learning nonlinear patterns *via* kernel tricks; however, its sensitivity to hyperparameter selection and data noise renders it somewhat less stable compared to XGBoost. Random Forest, a bagging-based ensemble method, excels at reducing variance but may fall short in modeling intricate patterns with the same precision as boosting-based algorithms. Lastly, although LightGBM offers rapid training through its leaf-wise growth strategy, it is prone to overfitting in relatively small or noisy datasets, which may have limited its ability to fully capture the underlying characteristics of the present data.

Given its consistently superior performance across evaluation metrics and stable convergence behavior during hyperparameter optimization, XGBoost was selected as the primary model for subsequent interpretability analysis using SHAP. The selection is further justified by its capacity to handle heterogeneous datasets and capture high-order feature interactions inherent in multi-factor leaching systems.

### 3.3 SHAP based feature importance analysis

**Fig. 6:**
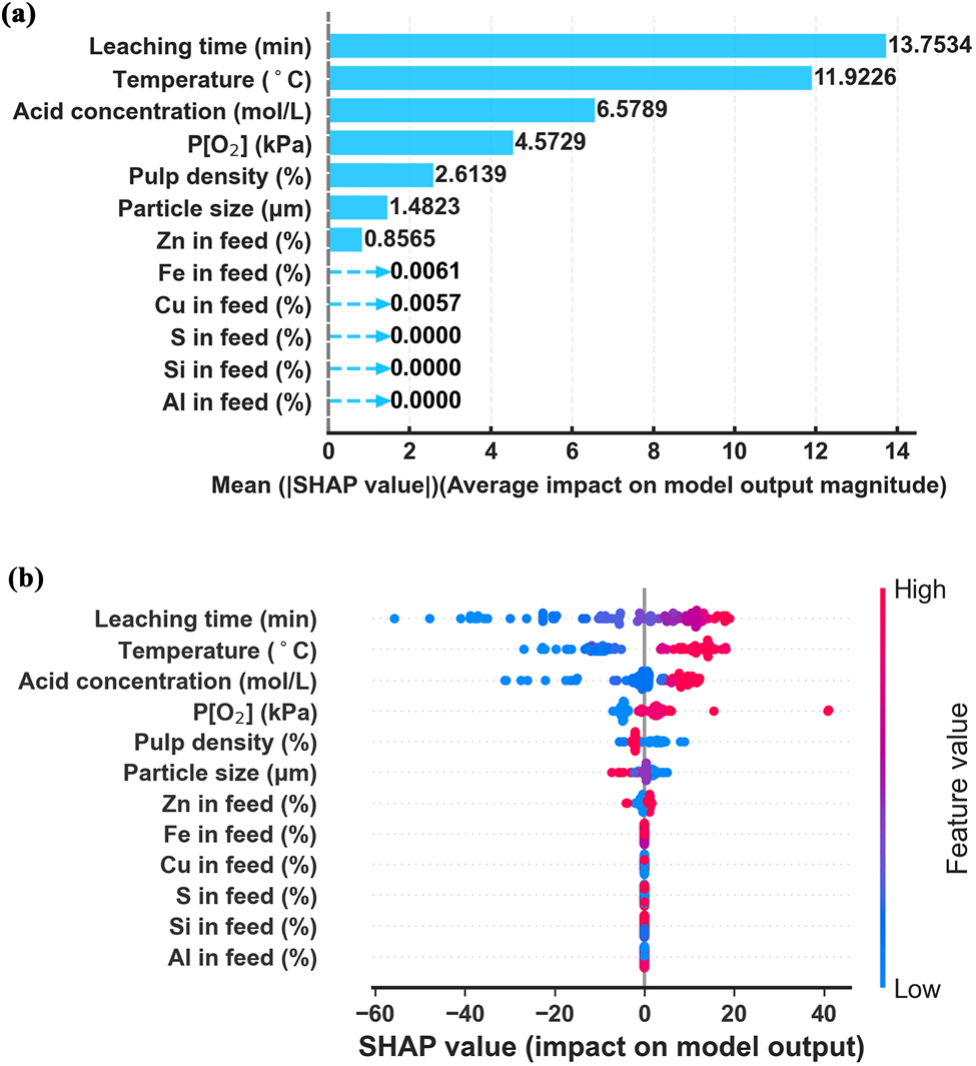
The SHAP analysis of the XGBoost model for (a) bar chart and (b) density scattering plot of feature importance.

**Fig. 7:**
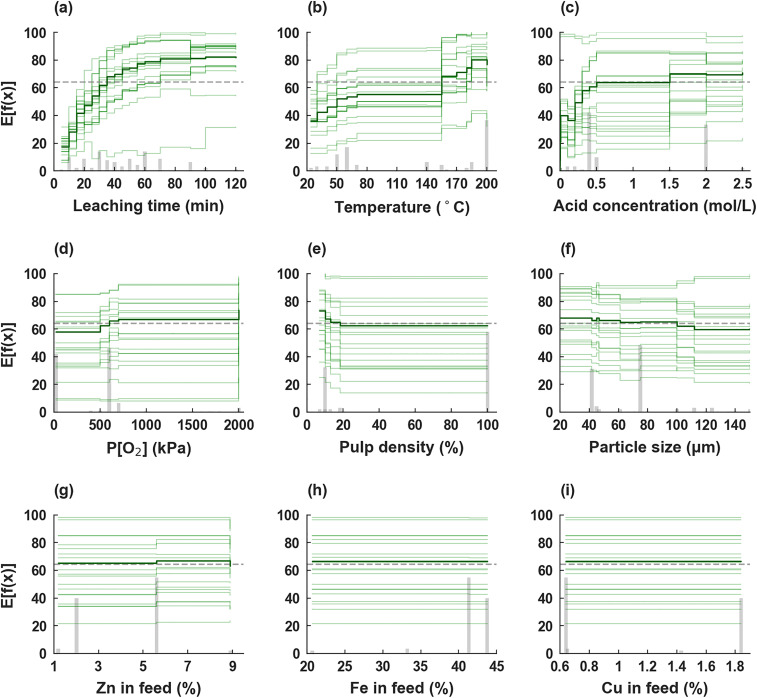
Partial dependence of SHAP analysis for the XGBoost model for copper leaching efficiency: (a) leaching time; (b) temperature; (c) acid concentration; (d) P[O_2_]; (e) pulp density; (f) particle size; (g) Zn in feed; (h) Fe in feed; (i) Cu in feed (%).

To elucidate the underlying driving factors governing copper leaching efficiency and enhance model interpretability, SHapley Additive exPlanations (SHAP) analysis was performed on the optimized XGBoost model. The global feature importance and feature-response relationships are presented in Fig. 6 and 7.

The global importance ranking based on mean absolute SHAP values is shown in Fig. 6a, while the SHAP summary plot is illustrated in Fig. 6b. The results indicate that operational parameters exert a stronger influence on copper leaching efficiency than compositional variables.

Among all input features, leaching time, temperature, and acid concentration emerge as the most influential variables. Oxygen pressure, pulp density and particle size also exhibit substantial contributions. In contrast, compositional variables such as Zn display moderate but comparatively smaller effects.

The dominance of leaching time, temperature, and acid concentration is consistent with reaction kinetics principles, as copper dissolution in sulfuric acid systems is governed by proton availability, temperature-dependent reaction rates, and reaction time progression. The SHAP results quantitatively confirm that process conditions outweigh intrinsic compositional variability within the investigated dataset.

Interestingly, Si, S, and Al contents exhibit zero mean SHAP values in the optimized XGBoost model, indicating minimal direct contribution to the prediction of copper leaching efficiency within the investigated dataset. This observation does not necessarily imply that these elements are chemically irrelevant; rather, it suggests that their effects may be secondary compared to dominant operational parameters such as temperature, acid concentration, and leaching time.

From a metallurgical perspective, the relatively weak contributions of Zn and Al can be rationalized by their typical occurrence and behavior in copper slag systems. Zinc is often present in oxide or spinel-type phases that dissolve relatively readily under acidic oxidative conditions, meaning that its presence does not significantly control the dissolution pathway of copper-bearing phases. Aluminum, on the other hand, is commonly associated with aluminosilicate structures that remain relatively stable in sulfuric acid leaching environments, thereby exerting limited direct influence on copper extraction kinetics.

From a statistical perspective, the compositional ranges of Zn and Al within the compiled dataset are relatively narrow compared with the broader variation of key operational parameters such as temperature, acid concentration, and leaching time. Machine learning interpretability methods such as SHAP therefore tend to assign lower importance to variables exhibiting limited variability or weaker correlations with the target response.

Therefore, the negligible SHAP contribution of Si, S, and Al should be interpreted as an indication of limited predictive relevance within the current dataset scope rather than complete mechanistic insignificance. Future studies incorporating mineralogical phase descriptors or broader compositional variability may further clarify their roles.

The partial dependence of various input variables on copper leaching efficiency is further elucidated through SHAP analysis, as illustrated in Fig. 7. As shown in Fig. 7a, leaching time exerts the most dominant positive influence, with a sharp increase in predicted efficiency observed within the initial 60 minutes of reaction. Beyond approximately 60 minutes, however, the marginal contribution gradually diminishes, exhibiting a saturating trend characteristic of the shrinking-core model. This behavior suggests that as the reaction progresses, increasing diffusion resistance becomes rate-limiting, thereby moderating the kinetic benefits of extended leaching time.

Temperature, acid concentration, and oxygen pressure also demonstrate clear positive correlations with the predicted leaching efficiency (Fig. 7b–d). Temperature exhibits a consistently positive contribution across its entire range, reaffirming its role as a fundamental kinetic driver in the dissolution process. Acid concentration, in contrast, displays a threshold behavior: leaching efficiency increases sharply up to approximately 0.5 mol L^−1^, beyond which further increases yield diminishing returns. This inflection point indicates a possible shift in the rate-controlling mechanism from surface reaction dominance to diffusion-limited kinetics once sufficient proton availability is secured.

Conversely, pulp density and particle size exhibit negative influences on the predicted leaching efficiency (Fig. 7e–f). Larger particle sizes and higher solid-to-liquid ratios both lead to reduced copper extraction, which aligns with fundamental hydrometallurgical principles concerning reduced specific surface area and impaired mass transfer characteristics.^37^

It is worth noting that the relative influences of oxygen pressure, pulp density, and particle size are considerably smaller in magnitude compared to those of leaching time, acid concentration, and temperature. This hierarchy of feature importance underscores the predominant role of operational parameters in governing leaching performance.

Among the compositional variables, the contents of Zn, Fe, and Cu in the feed material exhibit comparatively negligible effects on the predicted copper leaching efficiency, as shown in Fig. 7g–i. Their limited contribution relative to the dominant operational parameters suggests that, within the compositional ranges represented in the current dataset, process conditions outweigh intrinsic material variability in determining leaching outcomes.

Based on the SHAP partial dependence analysis (Fig. 7), recommended operating ranges for the primary process parameters were identified, as summarized in SI Table S3.

Overall, the SHAP analysis reveals that copper slag leaching efficiency is governed by a combination of proton-driven surface reactions, temperature-enhanced kinetics, diffusion limitations, and compositional resistance effects. Potential second-order interactions among key operational parameters were implicitly captured by the boosting structure, further supporting the model's capability to represent complex multivariate leaching dynamics. The machine learning model not only achieves high predictive accuracy but also captures mechanistically meaningful nonlinear behaviors consistent with established hydrometallurgical principles.

### 3.4 Validation and model generalization

To further evaluate the robustness and generalization capability of the optimized XGBoost model, the external validation datasets were selected from studies with partially distinct operating regimes to ensure rigorous generalization testing.^38,39^ This external validation strategy ensures that the predictive performance is assessed under truly unseen conditions, thereby providing a rigorous evaluation of model transferability.

**Fig. 8:**
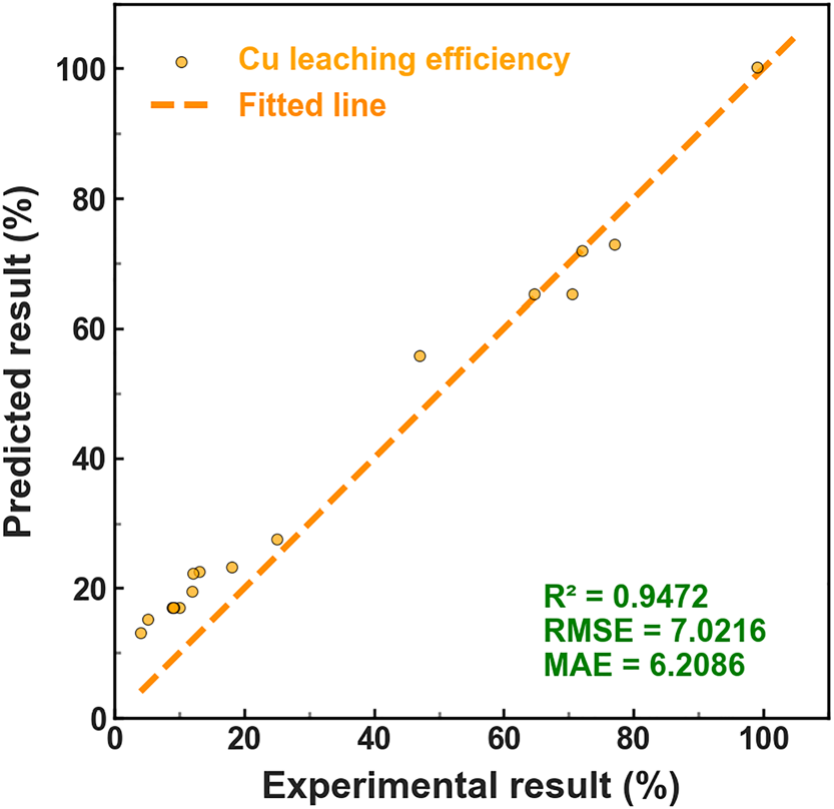
Verification performance for the XGBoost model of different cases.

The verification results are presented in Fig. 8. Predicted copper leaching efficiencies show strong agreement with experimentally reported values across both independent cases. The predicted-observed parity plots demonstrate close clustering around the 1 : 1 line, indicating minimal systematic deviation.

In both validation cases, the model accurately captures the trend of increasing leaching efficiency with rising temperature and acid concentration, as well as the influence of particle size and oxygen pressure. The consistency of prediction accuracy across distinct experimental regimes suggests that the model successfully learned generalized relationships rather than memorizing specific data patterns.

It is particularly noteworthy that the two external datasets encompass operating conditions partially different from the dominant regions of the compiled training dataset. Despite this variation, the model maintains stable predictive behavior, highlighting its ability to extrapolate within physically meaningful parameter ranges.

The strong performance under independent validation can be attributed to several factors. First, the compiled dataset spans wide compositional and operational domains, enhancing model exposure to diverse leaching scenarios. Second, the use of 10-fold cross-validation during hyperparameter optimization effectively mitigates overfitting. Third, the gradient boosting structure of XGBoost enables capture of nonlinear interactions and higher-order feature relationships that govern copper dissolution.

While the model demonstrates promising generalization performance, it should be noted that hydrodynamic conditions such as stirring speed were not included due to incomplete reporting in the literature. Therefore, predictions for systems with substantially different mixing regimes should be interpreted with caution.

Overall, the external validation results confirm that the developed XGBoost model is not merely a high-accuracy fitting tool but a robust and transferable predictive framework for copper slag leaching efficiency. The demonstrated generalization capability supports its potential application in process optimization and preliminary design evaluation in hydrometallurgical operations.

## 4 Conclusion

In this study, an interpretable machine learning framework was developed to predict copper leaching efficiency from copper slag under oxidative sulfuric acid conditions. Based on a systematically harmonized dataset of 465 experimental data points compiled from peer-reviewed literature, XGBoost was identified as the optimal model, achieving superior predictive accuracy with *R*^2^ = 0.9794, RMSE = 3.4757, and MAE = 2.3442 on the test set. SHAP-based interpretability analysis revealed that operational parameters, particularly leaching time, acid concentration, and temperature, exert dominant control over copper extraction, whereas the influence of compositional variables, including Fe, Zn, and Cu contents, remained comparatively negligible within the investigated dataset range. Particle size exhibited a negative contribution consistent with surface-area effects, and the observed nonlinear trends, including threshold behavior in acid concentration and saturating effects in leaching time, align well with shrinking-core kinetics and diffusion-controlled mechanisms. External validation using independent literature datasets confirmed the model's robust generalization capability across diverse operating conditions. This data-driven approach demonstrates that, under the investigated regimes, operational parameters outweigh compositional variability in determining leaching outcomes, thereby providing quantitative guidance for process optimization. Despite its robust predictive performance, this study has certain limitations that warrant future investigation. First, hydrodynamic parameters such as stirring speed were not incorporated due to inconsistent reporting in the original literature, which may influence mass transfer kinetics in reactor-specific applications. Second, the negligible SHAP contributions of Si, S, and Al should be interpreted within the compositional ranges of the current dataset; broader compositional variability or inclusion of mineralogical phase descriptors could further elucidate their roles. Future work should therefore focus on integrating hydrodynamic variables (*e.g.*, stirring speed) and expanding compositional diversity to enhance model generalizability and provide deeper mechanistic insights into copper slag leaching behavior.

## Ethical approval

This article does not contain any studies with human participants or animals performed by any of the authors.

## Author contributions

Sung-Jin Kim: conceptualization, methodology, software, validation, formal analysis, investigation, data curation, writing – original draft, writing – review & editing, visualization, supervision. Song-Sae Kang: methodology, software, formal analysis, data curation, writing – review & editing. Kyong-Nam Pae: investigation, data curation, writing – review & editing. Song-Il Pak: software, validation, writing – review & editing. Hyon-Il Jo: formal analysis, visualization, writing – review & editing. Ryong-Jin Kim: resources, supervision, writing – review & editing, project administration.

## Conflicts of interest

There are no conflicts to declare.

## Supplementary Material

RA-016-D6RA01571A-s001

## Data Availability

The data that support the findings of this study are available from the corresponding author upon reasonable request. The data were compiled from multiple peer-reviewed literature sources as cited in the manuscript, and the aggregated dataset used for model development is not publicly available due to ongoing research and potential for further analysis. Supplementary information (SI): hyperparameter optimization results (Fig. S1–S3), optimal hyperparameters for XGBoost, Random Forest, LightGBM, and SVR models (Table S1), model training time comparisons (Table S2), and recommended operating ranges for copper slag leaching parameters derived from SHAP partial dependence analysis (Table S3). See DOI: https://doi.org/10.1039/d6ra01571a.
