# Supplementary material for: Predicting copper leaching from slag: an interpretable machine learning approach under oxidative sulfuric acid conditions
Source: RSC Adv. 2026 Apr 13;16(21):19320–33. doi: 10.1039/d6ra01571a (PMC13071500; doi:10.1039/d6ra01571a)
Supplement: RA-016-D6RA01571A-s001 [file RA-016-D6RA01571A-s001.pdf]

Supplementary Information (SI) for RSC Advances.
This journal is © The Royal Society of Chemistry 2026

Predicting Copper Leaching from Slag: An Interpretable Machine Learning Approach under Oxidative Sulfuric Acid Conditions

Sung-Jin Kim[a,*], Song-Sae Kang[b], Kyong-Nam Pae[c], Song-Il Pak[b], Hyon-Il Jo[b], Ryong-Jin Kim[a]

[a] *Faculty of Materials Science, Kim Il Sung University, Pyongyang 497335, Democratic People's Republic of Korea*

[b] *Faculty of Information Engineering, Pyongyang HanTokSu University of Light Industry, Pyongyang 999093, Democratic People's Republic of Korea*

[c] *Faculty of Physical Engineering, Kim Chaek University of Technology, Pyongyang 950003, Democratic People's Republic of Korea*

[*] *E-mail: ksj1223@163.com*

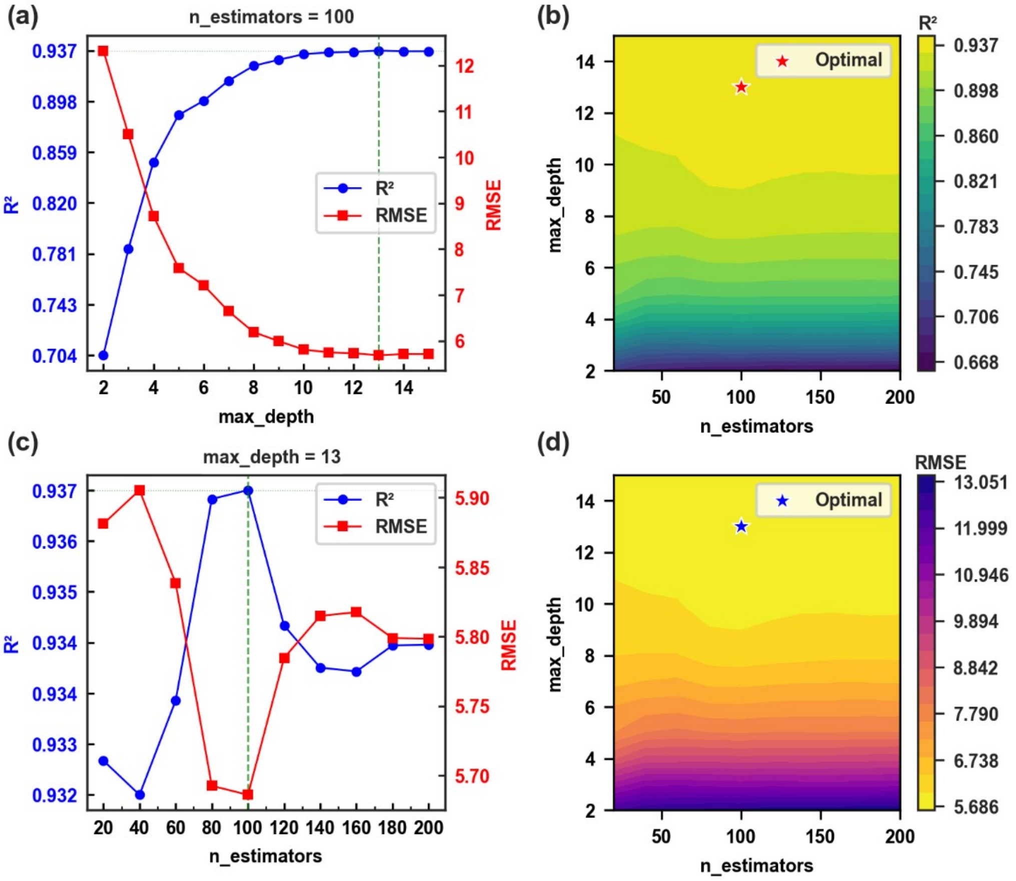

Fig.S1 (a) Optimization for the parameter of max_depth of the RF model; (b) Optimization for the parameter of n_estimator of the RF model; (c) Dependence of R^2 with the max_depth and n_estimators; (d) Dependence of RMSE with the max_depth and n_estimators

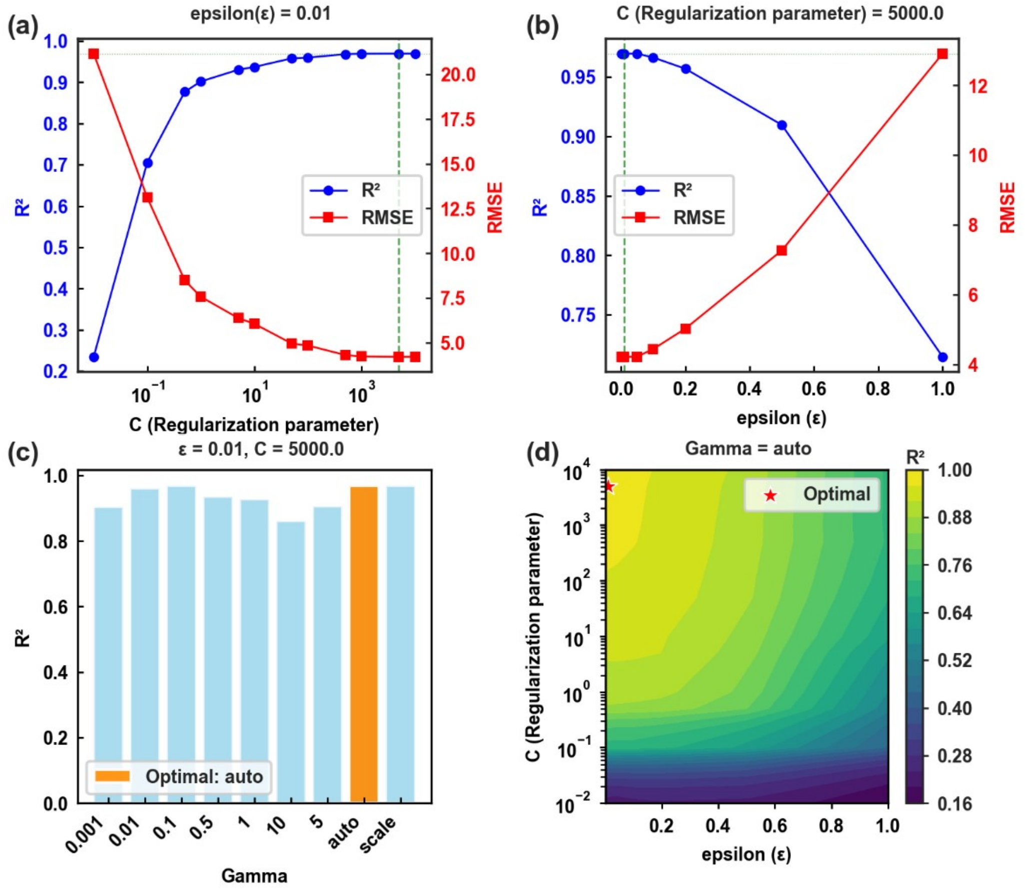

Fig.S2 (a) Optimization for the parameter of C of the SVR model; (b) Optimization for the parameter of epsilon of the SVR model; (c) Optimization for the parameter of gamma of the SVR model; (d) Dependence of R^2 with the C and epsilon

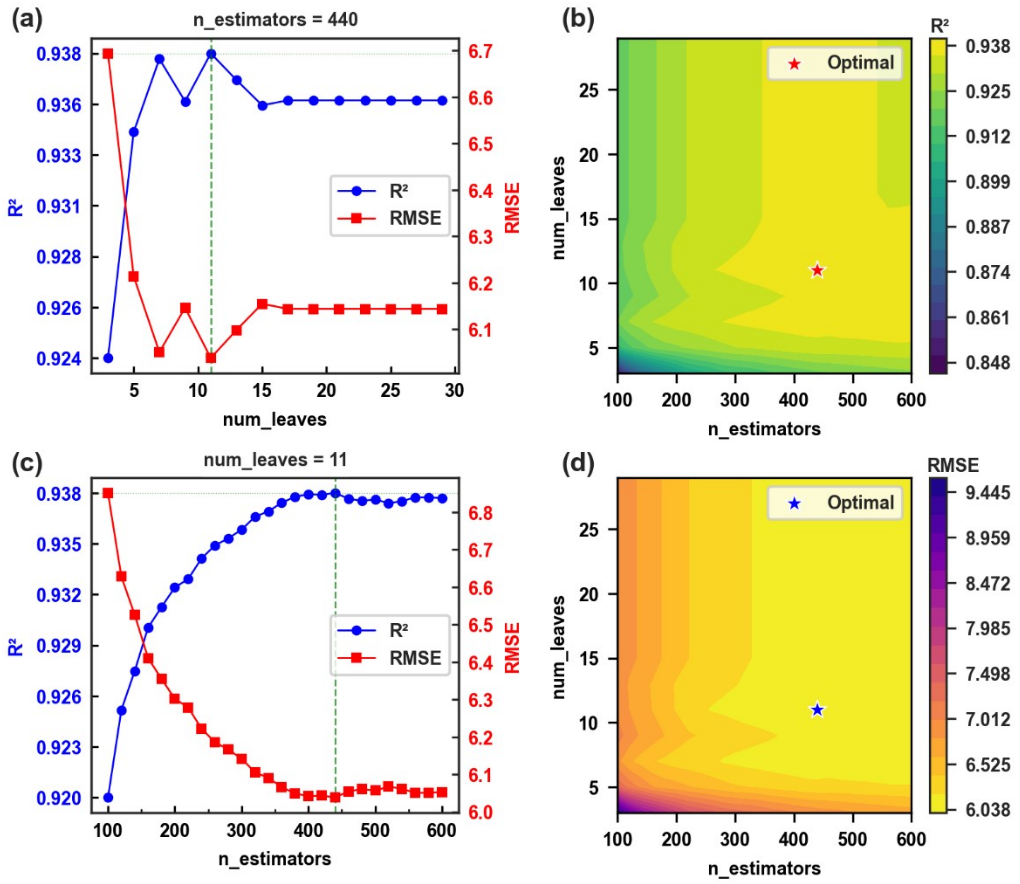

Fig.S3 (a) Optimization for the parameter of num_leaves of the LightGBM model; (b) Optimization for the parameter of n_estimator of the LightGBM model; (c) Dependence of R^2 with the max_depth and n_estimators; (d) Dependence of RMSE with the max_depth and n_estimators

Table S1. Hyperparameter search ranges and optimal values obtained through grid-search optimization.

Model	Hyperparameter	Search range	Optimal value
XGBoost	max_depth	2-15	4
	n_estimators	20-200	150
Random Forest	max_depth	2-15	13
	n_estimators	20-200	100
LightGBM	num_leaves	3-30	11
	n_estimators	100-600	440
SVR	C	0.01-10000	5000
	epsilon	0.001-1.0	0.01
	gamma	auto, scale, 0.001-10	auto

Table S2. Training times of the four machine learning models under identical

conditions.

Model	Training time (s)
XGBoost	0.53
Random Forest	0.51
LightGBM	0.33
SVR	1.26

Table S3. Recommended operating ranges for copper slag leaching parameters derived from SHAP partial dependence analysis of the XGBoost model.

Parameter	Recommended range	SHAP effect / interpretation
Leaching time (min)	40-80	Positive influence; SHAP increases rapidly then plateaus beyond 80 min
Temperature (°C)	120-160	Positive; monotonic increase, plateau at high end
Acid concentration (mol/L)	0.5-1.5	Positive; sharp increase below 0.5, plateau beyond 1.5
Oxygen pressure (kPa)	100-1000	Positive; effect diminishes above 1000
Pulp density (%)	5-30	Negative trend; lower pulp density favors efficiency
Particle size (μm)	<75	Negative trend; smaller particle size favors efficiency
